# Supplementary figures and images for: The impact of Ramadan fasting on glucose variability in type 2 diabetes mellitus patients on oral anti diabetic agents
Source: PLoS One. 2020 Jun 29;15(6):e0234443. doi: 10.1371/journal.pone.0234443 (PMC7323947; doi:10.1371/journal.pone.0234443)

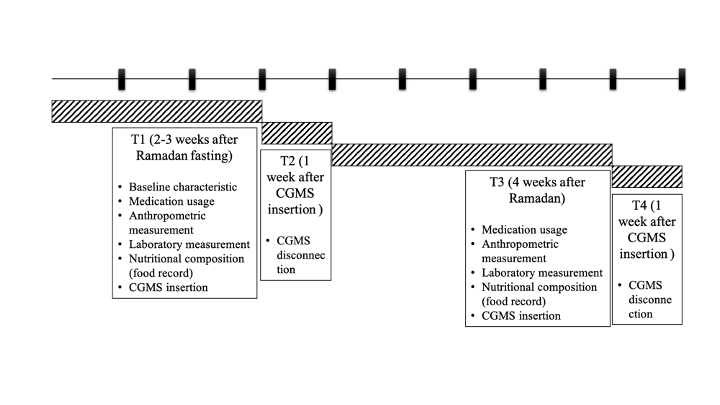

Supplement: S1 Fig — (TIFF) [file pone.0234443.s001.tiff]

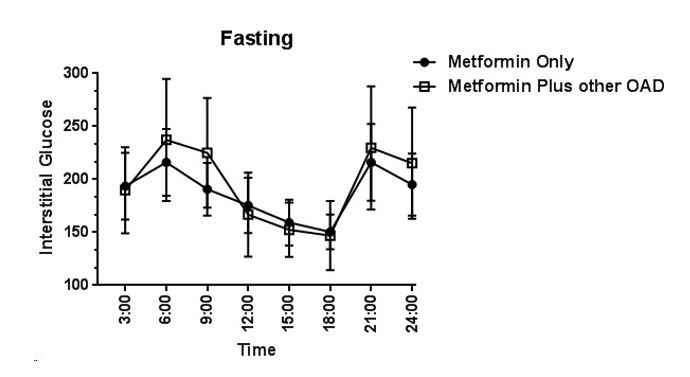

Supplement: S2 Fig — (TIF) [file pone.0234443.s002.tif]

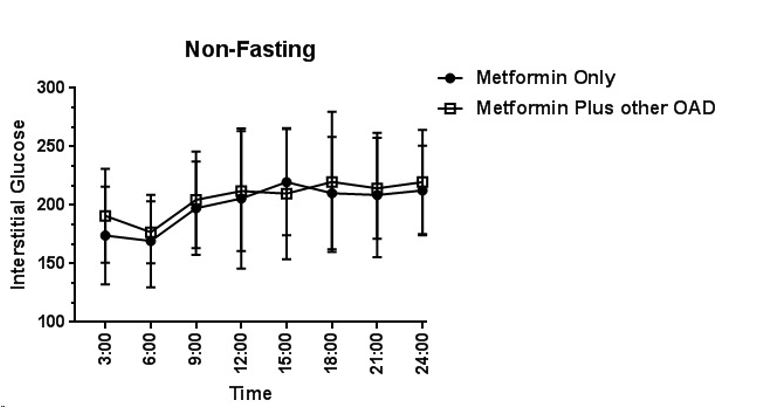

Supplement: S3 Fig — (TIF) [file pone.0234443.s003.tif]
