# Supplementary material for: The impact of Ramadan fasting on glucose variability in type 2 diabetes mellitus patients on oral anti diabetic agents
Source: PLoS One. 2020 Jun 29;15(6):e0234443. doi: 10.1371/journal.pone.0234443 (PMC7323947; doi:10.1371/journal.pone.0234443)
Supplement: S1 Table — (DOCX) [file pone.0234443.s004.docx]

S1 Table. Comparison of Dietary Compositions during Ramadan and after Ramadan

| **Diet Parameter** | **Ramadan** | **After Ramadan** | **p value** |
| --- | --- | --- | --- |
| Energy (kcal, mean, SD)  Carbohydrate (g, mean, SD)  Protein (g, mean, SD) | 1 455 (687)  191 (84)  46 (27) | 1 402 (315)  169 (25)  52 (14) | 0.79  0.21  0.17 |
| Fat (g, mean, SD) | 60 (32) | 60 (20) | 0.63 |
| Saturated Fatty Acid (g, mean, SD) | 13 (7) | 15 (3) | 0.32 |
| Mono-Unsaturated Fatty Acid (g, mean, SD) | 25 (13) | 23 (9) | 0.88 |
| Poly-Unsaturated Fatty Acid (g, mean, SD) | 16 (12) | 17 (6) | 0.20 |
| Cholesterol (g, mean, SD) | 117 (76) | 219 (57) | 0.58 |
| Fiber (g, mean, SD) | 13 (6) | 10 (4) | 0.84 |

Saturated Fatty Acid=SFA, Mono-Unsaturated Fatty Acid= MUFA, Poly-Unsaturated Fatty

Acid= PUFA. p value < 0.05 is considered statistically significant
