# Supplementary material for: The impact of Ramadan fasting on glucose variability in type 2 diabetes mellitus patients on oral anti diabetic agents
Source: PLoS One. 2020 Jun 29;15(6):e0234443. doi: 10.1371/journal.pone.0234443 (PMC7323947; doi:10.1371/journal.pone.0234443)
Supplement: S2 Table — (DOCX) [file pone.0234443.s005.docx]

**S2 Table. Comparison of Dietary Compositions during Suhur and Iftar**

| **Diet Parameter** | **Suhur** | **Iftar** | **p value** |
| --- | --- | --- | --- |
| Energy (kcal, mean, SD)  Carbohydrate (g, mean, SD)  Protein (g, median, IQR) | 568 (347)  79 (49)  17.5(14.4-20.1) | 897 (319)  112 (27)  24.6(18.2-34.9) | 0.07  0.09  0.05 |
| Fat (g, mean, SD) | 39 (18) | 21 (17) | 0.07 |
| Cholesterol (g, mean, SD) | 66 (72) | 79 (55) | 0.66 |
| Fiber (g, mean, SD) | 4.3 (2.6) | 7.5 (2.2) | 0.008 |

Saturated Fatty Acid=SFA, Mono-Unsaturated Fatty Acid= MUFA, Poly-Unsaturated Fatty

Acid= PUFA. p value < 0.05 is considered statistically significant
