# Supplementary material for: The impact of Ramadan fasting on glucose variability in type 2 diabetes mellitus patients on oral anti diabetic agents
Source: PLoS One. 2020 Jun 29;15(6):e0234443. doi: 10.1371/journal.pone.0234443 (PMC7323947; doi:10.1371/journal.pone.0234443)
Supplement: S3 Table — (DOCX) [file pone.0234443.s006.docx]

Table S3. Comparison of Interstitial Glucose during Ramadan and after Ramadan

| **Timepoint** | **Ramadan** | **After Ramadan** | **p value** |
| --- | --- | --- | --- |
| IG at 3 AM (mg/dL, median, IQR) * | 154.803 (131.54-252.45) | 168.717 (132.58-247.94) | 0.779 |
| IG at 6 AM (mg/dL, mean, SD) | 226.653 (79.583) | 173.171 (55.795) | 0.027 |
| IG at 9 AM (mg/dL, mean, SD) | 207.909 (71.418) | 201.041 (60.084) | 0.652 |
| IG at 12 AM (mg/dL, mean, SD) | 205.143 (80.760) | 216.155 (62.086) | 0.552 |
| IG at 3 PM (mg/dL, median, IQR * | 134.119 (100.18-220.46) | 201.04 (170.63-272.994) | 0.012 |
| IG at 6 PM (mg/dL, median, IQR) * | 125.753 (97.91-227.36) | 199.945 (175.97-276.22) | 0.012 |
| IG at 9 PM (mg/dL, mean, SD) | 222.872 (78.669) | 211.629 (54.291) | 0.558 |
| IG at 12 PM (mg/dL, mean, SD) | 171.047 (71.896) | 208.904 (58.786) | 0.114 |

Interstitial Glucose=IG, Standard Deviation=SD, p value < 0.05 is considered statistically significant

* with Wilcoxon Signed Rank Test
